# Chorioretinal venous anastomosis for non-ischemic retinal vein occlusion

**DOI:** 10.3389/fopht.2022.869843

**Published:** 2022-08-23

**Authors:** Yucheng Wang, Shaolong Wang, Shiwu Wang, Min Ding, Min Zhang, Jiannan Tang, Aijun Deng

**Affiliations:** ^1^ Eye Center of Affiliated Hospital of Weifang Medical University, Weifang, China; ^2^ Weifang Medical University, Weifang, China

**Keywords:** retinal vein occlusion, chorioretinal venous anastomosis, branch retinal vein occlusion, central retinal vein occlusion, treatment

## Abstract

Retinal vein occlusion (RVO) refers to the occlusion of the central retinal vein or primary and secondary branches caused by multiple factors. Clinical treatments for it include intravitreal or systemic vasodilator application, local usage of steroids and NSAID (non-steroidal anti-inflammatory drugs), thrombolysis, hemodilution, retinal laser photocoagulation, vitrectomy with vascular sheath incision, chorioretinal venous anastomosis (CRVA), and so on. At present, most treatments are aimed at RVO complications, while chorioretinal vein anastomosis can fundamentally reflux retinal vein blood through the choroid by venous vascular remodeling. Reports on the treatment of retinal vein occlusion by chorioretinal anastomosis are numerous in various countries. As a treatment means, CRVA can drain the venous blood, skipping the thrombosis spot, thus partially relieving anatomical vascular occlusion to achieve a therapeutic purpose. In this study, CRVA is evaluated from the aspects of indications, implementation process, postoperative effect evaluation, complications, and combination with anti-VEGF treatment. Based on the development of laser technology and vitrectomy, we hope to further review this treatment and provide a new reference for the clinical treatment of RVO.

## Introduction

Retinal vein occlusion (RVO) is the second-largest cause of visual impairment in retinal vascular diseases, following diabetic retinopathy. Its morbidity has reached 2.6%, and the prevalence among patients over 40 years old is about 1% to 2% (1). RVO is classified into Central Retinal Vein Occlusion (CRVO), Branch Retinal Vein Occlusion (BRVO), and Hemispheric Retinal Vein Occlusion (HRVO), with two subtypes including ischemic and non-ischemic lesions. A report on RVO by the Central Vein Occlusion Study Group (CVOS) found that 34% of the 714 study eyes that initially showed non-ischemic lesions developed ischemic lesions during the follow-up period (2). RVO is associated with many risk factors for RVO, including age, the crossing of retinal arteries and veins (AV), high intraocular pressure, short eye axis, retinal vascular wall sclerosis, dyslipidemia, hyperglycemia, hypertension, smoking, etc. Its etiology is complex and the pathogenesis is not fully understood (3–6). Currently, other than symptomatic treatments for RVO there is no fundamentally effective therapy available. In recent years, the chorioretinal venous anastomosis (CRVA) technology has been used for RVO. It can drain the middle retinal vein blood to the choroidal vein through the remodeling of retinal veins, establishing a new path of blood circulation and anatomically alleviating the ischemia caused by vascular occlusion and poor blood circulation.

## Brief introduction of chorioretinal vein anastomosis (CRVA)

Laser-induced chorioretinal venous anastomosis (L-CRVA) was confirmed by McAllister in 1992 through animal experiments (7), and has been gradually improved and applied in clinical practice to treat RVO. With the popularization of this technology, it has become a new means of RVO treatment and one of the most feasible means of clinical research. After CRVA was established, it was used in clinics in 1995 to treat non-ischemic RVO. Commonly used treatment methods for RVO, such as retinal laser photocoagulation and intravitreal injection of anti-Vascular Endothelial Growth Factor (anti-VEGF), are all aimed at the treatment of RVO complications, but cannot recanalize the occluded blood vessels. When RVO occurs, retinal venous circulation is blocked, which leads to retinal blood stasis and elevated central retinal venous pressure (CVP). Studies have shown that there is a collateral circulation between the retinal vein and the choroidal vein, through which the retinal vein blood can flow into the choroidal vein through the anastomosis of collateral circulation at a certain pressure difference between the retinal vein and choroidal vein, which provides a physiological basis for CRVA (8). Therefore, CRVA can prevent or delay the development of RVO from non-ischemic type to ischemic type by remodeling the blood vessels and changing the blood reflux pathway, and provides an opportunity for the application of other treatment methods (9).

## Indications for chorioretinal vein anastomosis (CRVA)

The damage of ischemia to the eyes with RVO is more serious than that of non-ischemia. In non-ischemic lesions, although there are different degrees of blood stasis, the capillary circulation of the retina remains intact, and the complications are mild. When ischemic lesions occur, the lack of perfusion of retinal capillaries is mostly accompanied by retinal ischemia and tissue necrosis, and complications such as retinal neovascularization, macular edema, and neovascular glaucoma evolve more frequently. Therefore, in the early diagnosis of non-ischemic RVO, timely removal of vascular obstruction and drainage of blocked blood flow by CRVA can improve the prognosis. However, for RVO with deficient blood perfusion, visual acuity and other prognostic treatments are poor even if blood circulation is restored (10, 11). Therefore, based on the prediction of the post-treatment effect, non-ischemic RVO is generally regarded as the preferred indication for CRVA treatment. After excluding anterior segment lesions and other ocular and systemic lesions that may affect the treatment, non-ischemic RVO patients are the target patients for CRVA treatment. The diagnosis of non-ischemic RVO is mainly based on direct fundus examination and fundus fluorescein angiography (FFA): ① Retinal veins are tortuous and dilated with patchy hemorrhage in each quadrant; ②FFA showed that the total area of the nonperfused retinal capillary was less than or equal to 10 optic disks.

## Implementation of chorioretinal vein anastomosis (CRVA)

CRVA can be performed by multiple means of implementation, including photochemical method, laser induction, and vitrectomy. The retinal vein, retinal pigment epithelium (RPE), and Bruch membrane are broken and connected to drain the blood. Different implementation methods may encounter different difficulties, among which laser-induced CRVA and vitrectomy CRVA are mostly used for clinical treatment.

### Chorioretinal anastomosis induced using photochemical method

The earliest method that can be applied to CRVA is the photochemical method. The report showed that the structure of the cell membrane can be destroyed by the chemical reaction between photons and photosensitizers (12). Therefore, the venous wall can be destroyed by this method to recanalize the drainage of venous collateral circulation. However, due to the incomplete clear related mechanism and unstable chemical reaction, it is difficult to apply in the clinical RVO treatment.

### Laser-induced chorioretinal anastomosis (L-CRVA)

Laser-induced chorioretinal anastomosis (L-CRVA) is a widely studied and viable treatment in the clinic, especially in the treatment of RVO. This method can alleviate vascular occlusion and reduce the elevated CVP due to the advantages of accurate location and high anastomosis recanalization rate. Before treatment, FFA and ICGA were operated to locate the occlusion site and the selected choroidal anastomosis site. When CRVO was performed, 2–3 disc diameter (DD) above or below the temporal or nasal side of the optic disc was selected, and BRVO was performed at a position 1DD away from the occlusion point and far away from the macula. L-CRVA can be performed by a single wavelength laser or combined with multiple wavelength lasers. Lu et al. (13) completed the operation and compared single wavelength laser, two-wavelength laser, and three-wavelength lasers, and advocated that the combination of various wavelength lasers can achieve a better therapeutic effect, which is consistent with the research results of McAllister et al. (14). When the two lasers were combined, the RPE layer, Bruch membrane and retinal vein wall were first broken by argon blue-green laser (2–2.4 W, 50 μm, 0.1 s) for one to two times, and the choroidal vein wall was broken by Nd YAG laser (a wavelength of 532 nm, 3–8 mj). When the three lasers were combined, the RPE layer, Bruch membrane and retinal vein wall were first broken by argon blue-green laser (1.6–2 W, 50 μm, 0.1 s) irradiation one to two times, then Krypton red laser (a wavelength of 647 nm, 1 W, 50 μm, 0.1 s) irradiation for one to two times at the same site; finally, the choroidal vein wall was broken by Nd YAG laser (3–8 mj) at the treatment site. The times of irradiation and the size of laser energy data can be adjusted according to the reaction degree of the breakdown site.

A new type of laser, Integre Plus (Ellex), was used in the research of McAllister et al. (15), which is equipped with a special mode solid-state laser photocoagulator and can provide up to 5 W of power at a wavelength of 532 nm. The reaction efficiency and degree between the laser and retinal tissue depend on the energy density and spot diameter of the laser, and the spot diameter is inversely proportional to the energy density. The Integre Plus (Ellex) laser has a smaller spot, and its energy density is 29% higher than that of traditional Nd YAG (HGM K3). When Integre Plus (Ellex) is used, it is equivalent to 71% of the power required by the HGM K3 laser, which can break down the retinal vein wall or Bruch membrane, and the treatment efficiency is higher (16). Integre Plus (Ellex) laser is commercially available, so Integre Plus (Ellex) laser is a favorable option for L-CRVA treatment implementation.

### Surgical chorioretinal venous anastomosis performed through vitreous surgery

FeKrat and DeJnan first proposed vitrectomy for CRVA in 1999 and performed it in a patient with ischemic CRVO. After vitrectomy, the retinal vein and Bruch membrane were punctured with a 20 G puncture needle at the corresponding points of six selected sites. CRVA was formed in five sites after four months of reexamination (17). Peyman et al. (18) performed CRVA in five cases after vitrectomy. One part in four quadrants was selected during the operation, and the retina and Bruch membrane were cut with a retinal knife at the corresponding position. One end of a 5/0 Mersilene thread was placed in the vein, and the other end was placed in the retinal incision to keep the incision open, ensuring communication between the retina and the choroid and promoting the formation of CRVA. After that, retinal photocoagulation (Panretinal photocoagulation, PRP) was performed to prevent or reduce complications. At follow-up, one patient dropped out due to failure to complete the follow-up, while 10 of the remaining 16 subjects developed a functional CRVA.

Vitrectomy combined with radial optic neurotomy (RON) is also a surgical treatment for RVO. The diameter of the optic nerve and central retinal blood vessels (arteries and veins) entering the sclera is 3.0 mm, and the diameter of the scleral ring passing through the sieve plate is 1.5 mm. Therefore, when pathological changes occur, tissue develops edema or inflammation, squeezing of the central blood vessel causes abnormal hemodynamics, thrombosis, etc., which also becomes the inducement of RVO and complications such as retinal hemorrhage and macular edema occur. Based on this mechanism, Opremcak et al. (19) advocated the theory of RON to treat RVO and treated 11 patients with CRVO, and two patients formed CRVA at the optic disc incision. The operation relieves the compression of the scleral cribriform plate, scleral ring and surrounding tissues on the optic nerve and central retinal blood vessels and forms part of CRVA through the incision, which plays the role of local blood stasis and drainage. A three-channel vitrectomy is used to remove the vitreous body. The tip of the MVR blade is placed on the edge of the avascular area on the nasal side of the optic disk. The ideal cutting effect is to cut the scleral cribriform plate and the surrounding scleral tissue in an isometric range and make one or two incisions.

The incision is close to but not in contact with the main blood vessels. The piercing depth is such that the MVR blade exceeds the widest part of the rhombic tip. The direction of incision and separation is roughly parallel to the distribution of the optic nerve fibers to minimize damage to the peripheral optic nerve fibers and prominent blood vessels. Additionally, vitrectomy can reduce various inflammatory factors in the vitreous cavity, the inflammatory response of retinal blood vessels, and control the development of the disease (20). García-Arumíi (21) and Nomoto (22) performed RON to treat RVO patients, and CRVA was found in some patients in the postoperative follow-up. This may be because the RON incision deeply communicated the lacuna between the retina and choroid. With healing, CRVA gradually forms, and the corrected visual acuity and the degree of macular edema regression of the patients are significantly better than those without CRVA. Therefore, RON surgery is also a method to form CRVA, which can relieve the pressure on the scleral sieve plate and scleral ring and promote the circulation and reflux of stasis blood by developing CRVA.

## Evaluation of CRVA formation (**Table 1**)

### CRVA formation

During CRVA, the most important thing is to communicate the retinal vein with the choroid (7, 9, 10, 13). There is no unified standard to judge whether the venous wall and Bruch membrane have been punctured during L-CRVA. The ophthalmic triple mirror is mainly used to assist in observing the fundus, which is judged by the signs during the implementation process (7, 9): ① Small bleeding spots can be seen when puncturing the retinal vein wall; ②The punctate bubbles can be seen when the Bruch film is broken down. When a laser irradiates the vein wall or the Bruch membrane, local contraction of blood vessels may occur. Sometimes it is necessary to wait for blood vessel expansion before observing whether the laser breaks the vein wall or the Bruch membrane. For S-CRVA, vitrectomy can visually see the position of the retinal vein and Bruch membrane, which is easy to communicate. During L-CRVA and S-CRVA, hemorrhage may occur when the retinal vein and Bruch membrane are ruptured. When there is a small amount of bleeding, self-limiting hemostasis can be achieved. When there is occasional bleeding, the eyeball can be pressurized by a three-sided mirror to increase intraocular pressure for hemostasis during the implementation of L-CRVA. In contrast, increasing perfusion pressure can reduce the bleeding during the execution of S-CRVA (18, 19, 21–23).

**Table 1:** Summary of outcomes of treatment with CRVA.

Author(Reference)Year	Study	Participants	FUT (Months)	Successful (Proportion)	Improved VA	Adverse Events
McAllister (23) 2009	CCS	L-CRVA (55 eyes) Control (58 eyes)	18	42 eyes (76.4%)	8.3 letters (significant differed from control group)	Neovascularity (18.2%)
Leonard (14) 2003	RS	L-CRVA (19 eyes)	48	19 eyes (100%)	five lines in 16 patients (84.2%)	localized preretinal fibrosis (26%)
Lu (13) 2007	RS	L-CRVA (85 eyes)	6	60 eyes (70.6%)	more than two lines	vitreous hemorrhage (5%)
Peyman (18) 1999	RS	Vitrectomy (seven eyes)	6	five eyes (71.4%)	more than two lines	vitreous hemorrhage and preretinal fibrosis (14.3%)
Koizumi (24) 2002	RS	Vitrectomy (seven eyes)	6	five eyes (71.4%)	more than two lines	NA
Opremcak (19) 2001	RS	RON (11 eyes)	5–12	two eyes (18.2%)	more than two lines in eight patients (72.7%)	iris neovascularization (18.2%), may be caused by the development of disease course
García-Arumíi (20) 2003	PIS	RON (14 eyes)	16	six eyes (42.9%)	more than one line in eight patients (57.1%); more than two lines in 6 patients (42.9%)	vitreous hemorrhage (7.1%) and subretinal hemorrhage (21.4%)
Sato E (25) 2008	RS	RON(10 eyes)	3	NA	0.2 logMAR in 6 patients (60%); remained the same in 4 patients (40%)	NA
McAllister IL (26) 2021	CCS	L-CRVA + anti-VEGF(58 eyes)	24	NA	11.46 letters compared with control group	NA
McAllister IL (15) 2016	RS	L-CRVA(33 eyes)	23	29 eyes (88%)	NA	NA

NA (not available) indicates that the author of the reference article has not done any research in this area.

### Analysis of therapeutic effect and complications of CRVA

The formation of functional CRVA is evaluated by FFA and Indocyanine green angiography (ICGA), which usually takes 2–6 weeks to form and is reviewed once a month so that related complications can be found in time. During the follow-up, a standardized protocol can be used to evaluate the FFA and ICGA results of enrolled subjects (including the control group and treatment group):

1. Changes in CRVA
2. The effectiveness of retinal vein circulation after CRVA drainage
3. The progress of retinal ischemia
4. The occurrence and development of various adverse events.

The evaluation results can be obtained more objectively, scientifically, and accurately through standardized comparison. McAllister et al. (27) divided 113 patients into the control group (58 cases) and treatment group (55 cases) through standardized evaluation. The treatment group underwent L-CRVA, of which 42 patients (76.4%) developed functional CRVA, which was much higher than the success rate of early CRVA (33%) (9). The effectiveness of CRVA in venous drainage was reflected in the improvement of visual acuity, with an average gain of 8.3 letters (*P = 0.03*) in the treated eyes compared with the control group during the 18-month follow-up period. After 18 months, the baseline visual acuity of the treatment group increased by an average of 11.7 letters versus the control group (*P = 0.004*). However, 9.6% of the treated eyes developed ischemic CRVO (*P = 0.33*). Ten treated eyes (18.2%) encountered neovascularization at the site of L-CRVA, and non-ischemic CRVO developed into ischemic CRVO, which may be related to the aggravation of CRVO. CRVA drainage was incomplete, causing the formation due to the increased level of VEGF in the inflammatory response after laser treatment and local vascular occlusion ischemia at the distal end of the treatment site. When exploring the influencing factors of the CRVA success rate, 55 patients were studied. It was found that younger age (*P = 0.03*), better baseline visual acuity (*P = 0.04*), no history of hypertension (*P = 0.001*), argon laser combined with Nd YAG laser (*P = 0.06*) could improve CRVA anastomosis rate, and the gender of patients, CRVA position, and relative distance to the optic disk had no significant effect on CRVA success rate (14). In the study of Leonard et al. (24), all the 19 eyes included were successfully anastomosed (100%) without ischemia, and five eyes (26%) had two anastomoses. During the 48-month follow-up, the Snellen visual acuity of 16 eyes (84%) improved from one line to 11 lines (with an average increase of five lines), with only five rats (26%) having localized preretinal fibrous proliferative membrane. Lu et al. (13) found that 60 eyes (70.6%) formed effective CRVA, and their visual acuity improved by more than two lines after 6 months of follow-up. In 25 eyes (29.4%) without anastomosis, there was no significant improvement in visual acuity, and retinal hemorrhage increased in five eyes. However, the complications were alleviated after 2 to 6 months of treatment. Three eyes (5%) developed severe neovascularization, which caused severe vitreous hemorrhage and selected vitrectomy. Overall, the therapeutic effect of L-CRVL was apparent, and the therapeutic effect of three laser combinations was better than that of two laser combinations.

In the study of direct CRVA for RVO through glass resection, five of the seven patients observed by Peyman et al. (18) developed CRVA. After 6 months of follow-up, the visual acuity of five patients improved by two lines. One patient developed vitreous hemorrhage and fibrous proliferative membrane, which needed further operations. In addition, one patient developed cataract aggravation after the operation, which could not be followed up. However, the cataract aggravation in this patient may be related to vitrectomy but not CRVA treatment. Koizumi et al. (25) studied seven patients who were treated with CRVA after vitrectomy combined with cataract surgery. The retina and Bruch membrane were cut on both sides of the retinal vein at the occlusion site, and the incision was surrounded by retinal laser photocoagulation after cutting off the vein. During the postoperative follow-up, CRVA was formed in five patients, and their visual acuity improved by at least two lines without macular edema. After repeated studies, Koizumi found that the success rate of CRVA was 71% higher than that of most related reports. The procedure was speculated to have unknown benefits for the recovery of diseased veins. RVO was first treated by RON (19). Eleven patients in the study were followed up for an average of 9 months (five cases for 12 months), and nine patients (82%) had improved or not decreased visual acuity after operation, and eight patients (73%) had rapidly improved visual acuity, with an average increase of five lines, which was speculated to be related to the decrease in perioptic pressure and the formation of circulatory bypass by CRVA. However, in the later follow-up, iris neovascularization occurred in two patients, which may not be related to the operation itself but the progression of the disease. After RON treatment, eight out of 14 (57.1%) patients with CRVO had visual acuity improved by at least one line, six patients (42.9%) had visual acuity improved by at least two lines (*P <0.001*), and macular thickness decreased significantly (*P <0.001*). The other six eyes (42.9%) had CRVA at RON incision with better corrected visual acuity, even though there was no statistical significance (*P = 0.28*), which may be related to the small number of cases. During postoperative observation, vitreous hemorrhage occurred in one case (7.1%), which was completely absorbed within 3 weeks, and subretinal hemorrhage occurred in three cases (21.4%), which were subsequently absorbed (21). Sato et al. (28) performed RON on 10 eyes of 10 CRVO patients (including two ischemic and eight non-ischemic) after vitrectomy. Compared with the average best-corrected visual acuity and average fovea thickness before and 3 months after operation, the average amplitudes of A wave and B wave did not change significantly through the analysis of standardized combined electroretinograms (ERGs).Still the B/A wave ratio increased significantly and the B wave incubation period shortened significantly in six eyes. The B/A ratio in ERGs is a good index to evaluate retinal ischemia (29) so its ratio increases, indicating that retinal blood circulation and function are obviously improved.

Additionally, there are also reports of definite complications in CRVA treatment. Bavbek et al. (30) followed up eight eyes (three eyes with CRVO and five eyes with BRVO) after L-CRVA for 5 years. Two eyes (25%) had CRVA, and another patient had severe vitreous hemorrhage and iris neovascularization when followed up for about a year, which led to neovascular glaucoma. When RON formed CRVA, Sakaguchi et al. (23) observed five eyes of five patients with HRVO. Through ICGA and perimetry evaluation, three eyes (60%) were found to have found retinal choroidal tumors and two eyes had temporal visual field defects.

### CRVA therapy and anti-VEGF therapy

Studies have found that VEGF plays a crucial role in RVO complications, particularly neovascularization and macular edema (31, 32). In recent years, anti-VEGF therapy has become a milestone treatment for RVO complications and can achieve satisfactory results for clinicians and patients (26, 33). However, a characteristic of RVO complications treated with anti-VEGF is the recurrence of the disease. Therefore, the intravitreal injection of various anti-VEGF drugs is mostly “PRN therapy.” The primary factor leading to repeated complications is that the blocked blood vessels are not recanalized, resulting in long-term lesions, and anti-VEGF drugs can only control complications by reducing the binding of VEGF to receptors. From the perspective of anatomical and pathological factors, CRVA treatment can still be tried, and it can be combined with anti-VEGF therapy, particularly for recurrent and refractory RVO complications such as macular edema and neovascularization, which may have better results than anti-VEGF treatment alone. The purpose is to solve the important anatomical problem of vascular occlusion by recycling retinal blood flow and to cure or reduce the recurrence of RVO complications as much as possible.

It is reported in the literature that CRVA combined with intravitreal injection of anti-VEGF drugs can achieve a better therapeutic effect for treating RVO. In a two-year observation study (34), the results of the control group with an intravitreal injection of rezumab alone and the treatment group with an intravitreal injection of rezumab combined with L-CRVA in the PRN stage were compared. It was found that the average injection load of rezumab in the combined treatment group was 2.18 times (1.57 times to 2.78 times) in the first year. It was significantly lower than the average load of the control group by 7.07 times (6.08 times to 8.06 times) (*P <0.0001*). In the second year, the average load of the combined treatment group decreased to 0.94 times (0.62 times to 1.42 times), while the average load of the control group was 4.61 times (3.87 times to 5.47 times) (*P <0.0001*). The visual acuity of the treatment group significantly improved and the thickness of the macular area decreased significantly. Using logistic regression analysis, compared with the control group, the probability of “high CVP” in the treatment group decreased by 82.5% (*P <0.0001*). Therefore, CRVA combined with anti-VEGF drug therapy can better control complications and reduce the number of injections and economic burden on patients.

### Management of complications of CRVA

When implementing CRVA (including L-CRVA and S-CRVA), we should pay attention to the fact that the formation of CRVA may be accompanied by neovascularization around the optic disc and anastomosis, vitreous hemorrhage, neovascular glaucoma, localized retinal proliferative membrane, etc. Patients with abnormal coagulation function are more prone to related complications, so the complications of CRVA cannot be ignored. During the treatment of CRVA, attention should be paid to the occurrence of complications, and corresponding treatment should be done after timely detection. Bleeding during CRVA is mostly self-limiting, but patients with abnormal coagulation function should consider the influence of coagulation function on the treatment effect and postoperative complications when choosing treatment, and even if they cannot be treated in this way for the time being, preoperative evaluation cannot be ignored. Common hemorrhage can be observed or treated conservatively with drugs. Severe vitreous hemorrhage can be observed conservatively for about 4 weeks. Vitrectomy is feasible if it cannot be absorbed by itself. CRVA anastomosis can be performed again during the operation. The more common localized retinal proliferative membrane is caused by an inflammatory reaction when a laser destroys the vascular wall structure. No special treatment is needed if there are no obvious signs such as proliferation, expansion, and traction during observation and follow-up. For the proliferative membrane that leads to proliferation, expansion, and traction, vitreous laser or vitrectomy can be used to release the traction. In terms of neovascularization and related complications, the typical choroidal neovascularization at the anastomosis is associated with Bruch’s membrane rupture (35). It is sensitive to anti-VEGF drugs, but the formation of CRVA may depend on the presence of VEGF. Therefore, without special circumstances, intravitreal injection of anti-VEGF drug to treat anastomotic neovascularization or combined with CRVA to treat RVO should not be performed prematurely. It is usually performed one month after discovering neovascularization (36) or with the combination of PRP.

## Summary

RVO is a common fundus disease in clinics. When RVO is diagnosed in one eye, the morbidity of RVO in the other eye within 4 years is about 7% (37). Studies have confirmed that the occurrence of retinal neovascularization is about 36% when the diameter of the non-perfusion area is larger than 5 DD (38), so diagnosis and treatment should be carried out in time. A series of research reports and data evaluations show that CRVA is an effective way to treat RVO, and its main indications are non-ischemic RVO lesions. L-CRVA and S-CRVA are commonly used clinical treatments. When RVO occurs, optical coherence tomography angiography (OCTA) shows that the perfusion pressure of the deep capillary plexus (DCP) in the macular area is lower than that of the shallow capillary plexus, and it is more likely to cause stasis and hypoxia damage due to the increase in CVP (39). The continuously increased CVP may increase the negative pressure in the DCP, resulting in progressive hypoxia, macular injury, and edema. CRVA can achieve recanalization or partial recanalization after retinal vein occlusion by forming a new venous drainage to reduce CVP, further reducing hemorrhage of retinal vessels and mitigating complications such as macular edema.

S-CRVA has higher costs, requires patients to have better cooperation and surgical tolerance, and more careful indications, more postoperative complications than L-CRVA, and is less popular in clinics. The advantages of L-CRVA include lower cost, less relative injury, lower cooperation requirements for patients, the exact effect and more applications, which is also the focus of research into non-ischemic RVO treatment. The treatment of CRVA can be repeated according to the postoperative reaction and disease development, and it is performed one to eight times in the literature. CRVA treatment can prevent or delay the development of non-ischemic lesions to ischemic lesions, but it cannot completely alleviate hypoperfusion. Although most reports of CRVA for treating RVO complications are ideal, serious complications cannot be ignored. Therefore, postoperative follow-up is critical, during which changes in CRVA and the timing of repeated treatment can be known and treated in time.

In recent years, clinicians have widely recognized anti-VEGF drugs for treating ROV complications. It has become the mainstream first-line treatment due to its high security, sound effects, and high compliance and acceptability among patients. However, treating repeated and refractory RVO complications is still tricky, and the simple application of anti-VEGF cannot meet the current treatment situation. For clinical and scientific researchers, finding a fundamental solution to vascular occlusion is still needed. We believe that CRVA, as one of the viable treatments, deserves our in-depth exploration. Although in the past, due to unstable equipment and technology, CRVA was difficult to implement, with slow research progress. However, with the improved stability and safety of the ophthalmic laser and the further improvement of vitrectomy equipment, CRVA is relatively safe and effective in treating RVO, especially non-ischemic lesions. However, careful case screening and strict follow-up are needed to achieve the best therapeutic effect and the slightest postoperative complications. CRVA combined with anti-VEGF drug therapy also provides another reference and method for repetitive and refractory RVO complications.

## Author contributions

All authors listed have made a substantial, direct, and intellectual contribution to the work and approved it for publication.

## Conflict of interest

The authors declare that the research was conducted in the absence of any commercial or financial relationships that could be construed as a potential conflict of interest.

## Publisher’s note

All claims expressed in this article are solely those of the authors and do not necessarily represent those of their affiliated organizations, or those of the publisher, the editors and the reviewers. Any product that may be evaluated in this article, or claim that may be made by its manufacturer, is not guaranteed or endorsed by the publisher.
